# Electrical conductivity and thermodynamic studies on Sodium Dimethyldithiocarbamate in non aqueous solvents Dimethylformamide (DMF), at different temperatures

**DOI:** 10.1038/s41598-022-18849-7

**Published:** 2022-09-17

**Authors:** W. A. Hammad, N. H. El-Hammamy, M. H. Morshidy, Kholood Alkamis, M. A. Darweesh

**Affiliations:** 1 Faculty of Engineering, Tanta University, Tanta, Egypt; 2 Faculty of Science, Alexandria University, Alexandria, Egypt; 3 Tabuk University, Tabuk, Saudi Arabia

**Keywords:** Biochemistry, Environmental sciences, Chemistry, Energy science and technology, Engineering, Materials science, Physics

## Abstract

This paper threw some light on the behavior of Sodium *N*,*N*-Dimethyldithiocarbamate as an electrolyte. The effect of solvents on the conductance of this salt would be discussed via measurements of Λ_o_, a_o_ and KA, since it can be assumed that the different solvents have a little chance to impose great variations on the solvation processes. The conductance method was chosen as a tool to illustrate the electrolyte-solvent interactions. Fuoss–Onsager equation would be tested using Sodium Dimethyldithiocarbamate in presence of dimethylformamide solvent at different temperatures. The conductance of dilute solutions of Sodium *N*,*N*-Dimethyldithiocarbamate is measured in Dimethylformamide, at different temperatures (25, 30, 35 and 40 °C). Accurate values of **Λ**_o_ were obtained by applying the (Fuoss–Kraus–Shedlovsky) equation. Finally, the (Fuoss–Onsager) equation was solved to give the correct values of the constants **Λ**_o,_ J, K_A_ and a° (the closest distance of approach) for Sodium *N*,*N*-Dimethyldithiocarbamate salt in Dimethylformamide solvent.** Λ**_**o**_ and a° (solvation) increase with increasing temperatures. Thermodynamic parameters (∆G°, ∆H°, ∆S° and ∆E_s_) of Sodium *N*,*N*-Dimethyldithiocarbamate in Dimethylformamide were calculated from conductance measurements, the activation energy (∆E_s_), the enthalpy change (heat of association) (∆H°) and the entropy change (∆S°) are positive, however The free energy change (∆G°) values was negative for Sodium *N*,*N*-Dimethyl dithiocarbamate in DMF systems studied with increasing the temperature.

## Introduction

The density is one of the thermodynamic properties of electrolyte solutions and the viscosity is the transport properties of electrolyte solutions. Both dispensable basic data to design of engineering and process optimization.

Sodium *N*,*N*-dimethylthithiocarbamate's are important organic compound used in many applications and uses as a disinfectant, corrosion inhibitor, coagulant, vulcanizing agent, chelating agent, and fungicide which may result in its release to the environment^1^. Sodium *N*,*N*-dimethylthithiocarbamate's used in water treatment, rubber industry and is a registered biocide for cutting oils and aqueous systems in industries such as leather tanning and paper manufacturing^2^. Also it used as an antimicrobial agent in paints^3^.

In general, thermodynamic and transport properties of electrolyte solutions have a great importance due to their wide applications in different chemical industries (e.g. electrochemical process like corrosion or electrolysis, environmental applications, hydrometallurgical process, separation techniques like seawater desalination, crystallization and extractive distillation, and production of energy sources. Measuring the transport properties as conductance at low concentrations helps in exploring the ionic solvation also helps in obtaining reliable values of conductance at infinite dilution, these properties are affected by size of ions, any modulation in the structure of the solvent and the strong ion–solvent interactions^1,4,5^.

The aim of this paper is to explain the behavior of Sodium *N*,*N*-Dimethyldithiocarbamate as an electrolyte. The effect of solvents on the conductance of this salt would be discussed via measurements of Λ_o_, a° and K_A_, since it can be assumed that the different solvents have a little chance to impose great variations on the solvation processes. The conductance method was chosen as a tool to illustrate the electrolyte–solvent interactions. Fuoss–Onsager equation would be tested using Sodium Dimethyldithiocarbamate in presence of Dimethylformamide solvent at different temperatures (25, 30, 35 and 40 °C).

In addition, The variation of K_A_ in Dimethylformamide solvent was explained, the electrostatic radius of the ions was calculated using the Stokes' equation and a comparison between the sum (R^+^ + R^−^) and the closest distance for approach between cation and anion (a°) would be discussed and the activation energy ∆E_s_ and Thermodynamic parameters (∆G°, ∆H°, and ∆S°) were calculated in Dimethylformamide solvent at different temperatures (25, 30, 35 and 40 °C).

## Experimental

### Materials

**Figure 1:**
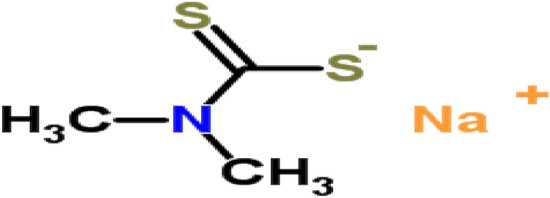
The structure of Sodium *N*,*N*-Dimethyl dithiocarbamate^10^.

Salt was highly purified grade and used without extra purification. Where Sodium *N*,*N*-Dimethyl dithiocarbamate (NaS_2_CN (CH_3_)_2_) is Analar analytical reagent "BDH" as shown in Fig. 1.

All glassware used were left over night in chromic acid, then washed with tap water, distilled water, conductivity water and finally steamed for about half an hour, and dried in an electric oven for 24 h.

#### Conductivity of Dimethylformamide

Analar analytical reagent Dimethylformamide (BDH), was used without further purification. The specific conductance _**°**_ for Dimethylformamide was found to be (1.6–2.6 × 10^–6^) ohm^−1^ cm^−1^.

### Apparatus and procedure

#### Preparation of solutions and salt

All solutions were prepared by weight. Salt is weighted by difference on a microbalance which reads to ± 0.1 mg. Dilution was carried out successively into the cell by siphoning the solvent by means of a weighing pipette. The salt was dissolved by carefully shaking the conductivity cell which is suspended in the water ultra-thermostat. The concentration of the solution was obtained by using the equation:

1 $$ C = \frac{{{\text{Wight}}\,{\text{of}}\,{\text{the}}\,{\text{salt }}}}{{{\text{equivalent}}\,{\text{wight}}\,{\text{of}}\,{\text{the}}\,{\text{salt }}}} \times \frac{{{\text{absoluted}}}}{{{\text{wight}}\,{\text{of}}\,{\text{solution}}}} \times 100\,{\text{eq}}./{\text{liter}}{.} $$

#### Conductance measurements

The cell constant was 0.1 cm^−1^ for dilute solutions. The measurement error of the conductivity meter used (± 1 digit) ≤ 0.5% and the reproducibility (± 1 digit) ± 0.1%.

The resistance of the solution was measured several time intervals (15 min) after throughly shaking.

The specific conductance of solute $\vartheta$ could be calculated from the relation:

2 $$ \vartheta = K\left( \frac{1}{r} \right)_{solution} , $$

where r is the resistance of solution, $\vartheta$ is the specific conductance of solute and could be calculated from the relation.

Different concentrations (C equiv. L^−1^) were prepared inside the cell using the weighing pipette as described before and the corresponding equivalent conductance values Λ, were calculated from the following equation:

3 $$ \Lambda = \frac{1000\vartheta }{C}, $$

where Λ is the equivalent conductance (ohm^−1^ equiv.^−1^ cm^2^), $\vartheta$ is the specific conductance of solution (ohm^−1^ cm^−1^), C is the concentration (equiv. L^−1^).

Precise conductance measurements were repeated for Sodium *N*,*N*-Dimethyldithiocarbamate in Dimethylformamide (DMF) at different temperatures (25, 30, 35 and 40 °C). The chemical formula for this salt is [(NaS_2_CN (CH_3_)_2_)].The abbreviations of this salt is (Na.DMDTC).

## Results and discussions

### Conductance measurements in pure solvent

#### Conductance of Sodium *N*,*N*-Dimethyldithiocarbamate in Dimethylformamide, at 25, 30, 35 and 40 °C

##### Application of equation of Fuoss–Kraus–Shedlovsky (F.K.S)

The solvent parameters of Dimethylformamide (DMF) at 25, 30, 35 and 40 °C, used in the calculations, are shown in Table 1^6–9^ where d is the absolute density at 25, 30, 35 and 40 °C, η is the viscosity at 25, 30, 35 and 40 °C, D is the dielectric constant at 25, 30, 35 and 40 °C.

**Table 1:** Solvent parameters of Dimethylformamide at 25, 30, 35 and 40 °C and 1 atm.

Temp (°C) ± 0.01	Absolute density (d) g/cm^3^	Dielectric constant (D)	10^2^ Viscosity (η) poise	Specific conductance () Ω^−1^ cm^−1^
25 °C	0.9443	36.70	0.8000	(1.4–1.8 × 10^–6^)
30 °C	0.9397	35.88	0.7535	(1.1–1.9 × 10^–6^)
35 °C	0.9350	34.95	0.7070	(1.3–1.7 × 10^–6^)
40 °C	0.9304	34.015	0.6680	(1.1–1.6 × 10^–6^)

Table 2 shows the $\vartheta$ is the specific conductance of the pure solvents at different temperatures. The values of equivalent conductance Λ (ohm^−1^ equiv^−1^ cm^2^) corresponding to several values of concentration C in (equiv. per liter) were obtained for the salt.

**Table 2:** Conductance of Sodium *N*,*N*-Dimethyldithiocarbamate in Dimethylformamide at 25, 30, 35 and 40 °C and 1 atm.

10^4^ C	C ^1/2^	10^6^ $\vartheta$	Λ
25 °C			
21.736	0.0466	131.14	60.332
19.964	0.0447	121.54	60.881
18.470	0.0430	113.34	61.365
17.218	0.0415	106.34	61.761
16.118	0.0401	100.14	62.128
15.149	0.0389	94.64	62.472
14.251	0.0378	89.44	62.760
13.451	0.0367	84.84	63.075
**30 °C**			
18.158	0.0426	124.98	68.829
16.759	0.0409	115.78	69.085
15.570	0.0395	107.98	69.353
14.565	0.0382	101.28	69.536
13.677	0.0370	95.38	69.737
12.890	0.0359	90.08	69.885
12.156	0.0349	85.18	70.071
11.499	0.0339	80.78	70.247
**35 °C**			
16.830	0.0410	119.11	70.771
15.522	0.0394	110.41	71.133
14.410	0.0380	103.01	71.484
13.473	0.0367	96.71	71.781
12.645	0.0356	91.11	72.051
11.912	0.0345	86.11	72.288
11.230	0.0335	81.41	72.496
10.619	0.0326	77.21	72.710
**40 °C**			
16.230	0.0403	114.85	70.765
14.926	0.0386	106.55	71.388
13.824	0.0372	99.45	71.941
12.899	0.0359	93.45	72.449
12.085	0.0348	88.05	72.861
11.366	0.0337	83.25	73.244
10.699	0.0327	78.75	73.603
10.104	0.0318	74.75	73.978

**Figure 2:**
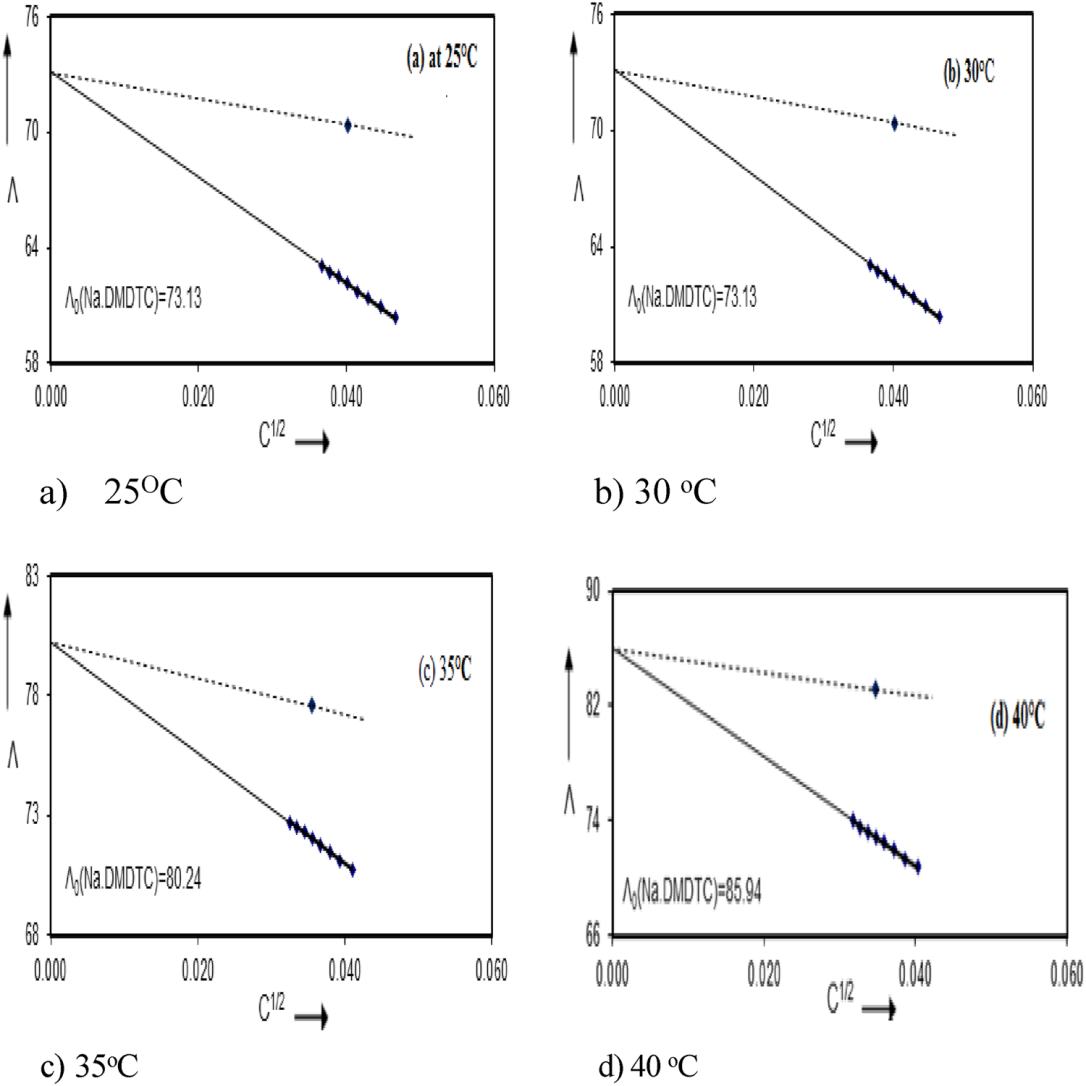
Conductance of Sodium *N*,*N* Dimethyl dithiocarbamate in Dimethylformamide at (**a**) 25, (**b**) 30, (**c**) 35 and (**d**) 40 °C.

**Figure 3:**
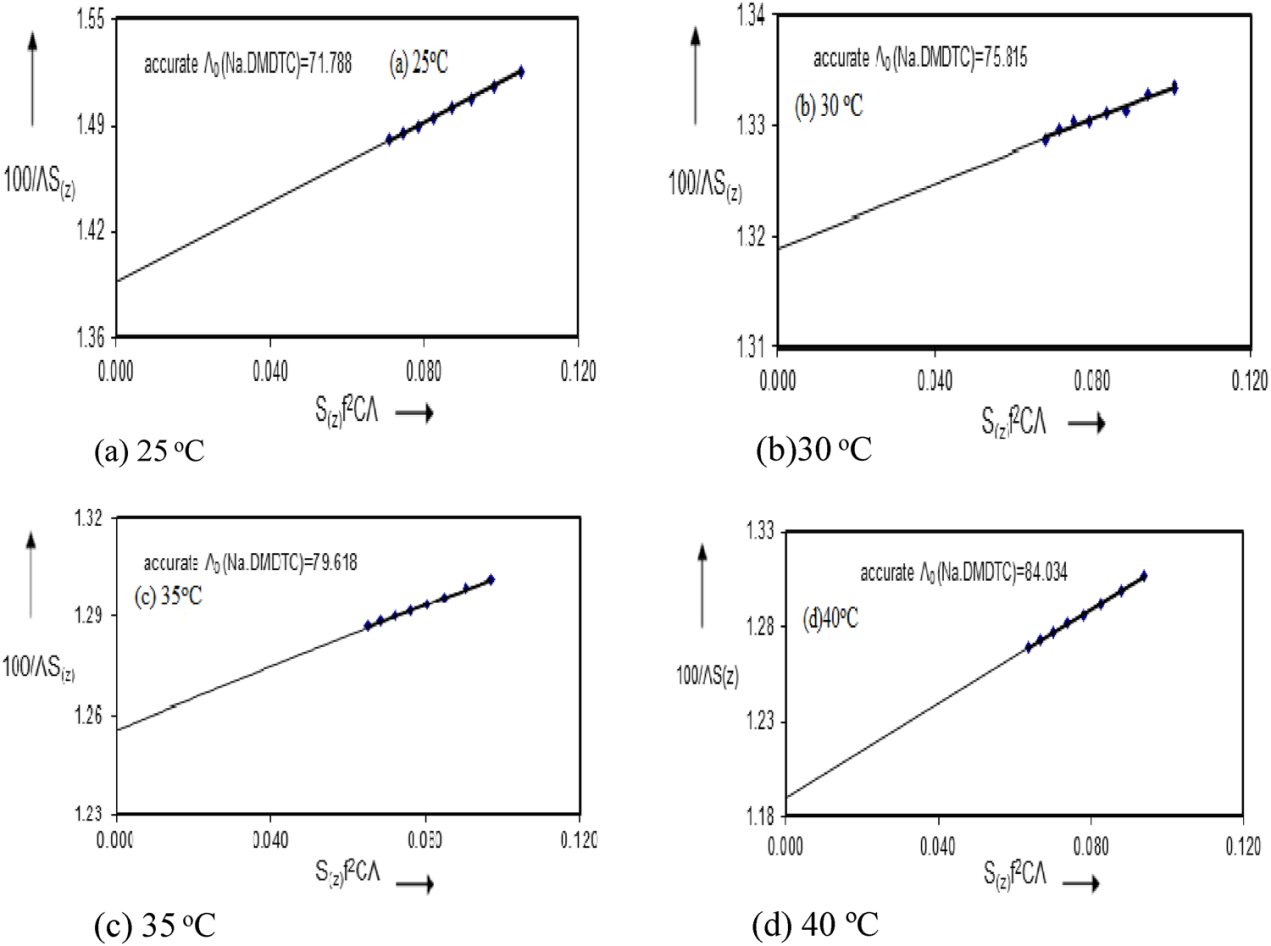
(F.K.S) plots for Sodium *N*,*N*-Dimethyl dithiocarbamate in Dimethylformamide at (**a**) 25 °C, (**b**) 30 °C, (**c**) 35 °C and (**d**) 40 °C.

A preliminary value of Λ_o_ (the equivalent conductance at infinite dilution) was estimated from Λ versus C^1/2^ plot as illustrated in Fig. 2. Accurate value of Λ_o_ was obtained from the (F.K.S) equation. Since a plot of 1/ΛS_(z)_ versus S_(z)_ f^2^CΛ as illustrated in Fig. 3 for DMF at 25, 30, 35 and 40 °C respectively should yield a straight line, the intercept then equals 1/Λ_o_ and the slope is 1/K_D_ Λ_o_^2^.

##### Application of Fuoss–Onsager equation:

The three-parameter shown in the Eq. (4):

4 $$ \Lambda ^{\prime }  = {\mkern 1mu} \Lambda _{{\text{o}}}  - {\text{ S}}({\text{c}}\gamma )^{{1/2}}  + {\text{ E }}\left( {{\text{c}}\gamma } \right){\text{ log }}\left( {{\text{c}}\gamma } \right){\mkern 1mu}  + {\text{J }}\left( {{\text{c}}\gamma } \right){\mkern 1mu}  - {\text{ K}}_{{\text{A}}} \left( {{\text{c}}\gamma } \right){\mkern 1mu} \Lambda {\text{ f}}^{2} .  $$

This theory was confirmed by the approximate agreement of ion sizes computed from Λ_o,_ J_(a°)_ and K_A_.

The calculated values of the parameters Λ_o,_ J_(a°)_ and K_A_ were calculated using a computer program. The accuracies which is represented by the standard deviation values are ± 0.02 for Λ_o,_ ± 2 for J < 200, ± 5 for J (200–1000) and ± 10 for J > 1000.

**Figure 4:**
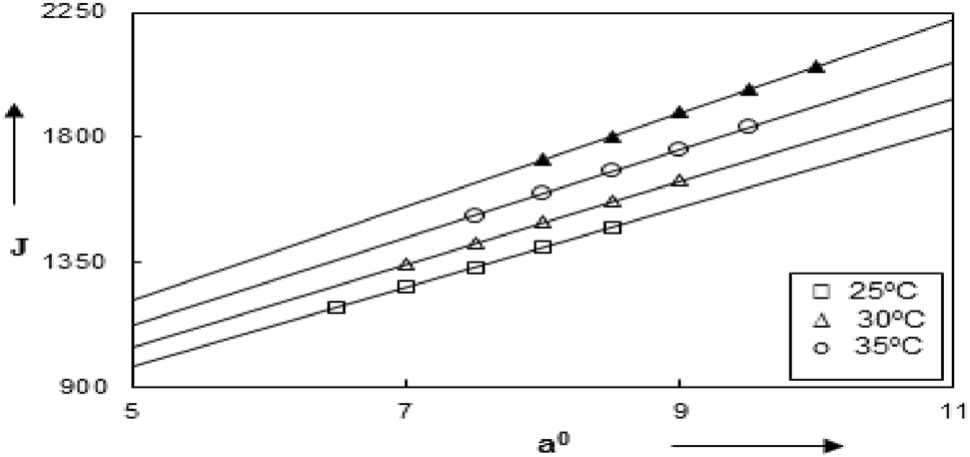
Variation of J and a° of Sodium *N*,*N*-Dimethyl dithiocarbamate in Dimethylformamide at 25, 30, 35 and 40 °C.

Figure 4 show the variation of a° with J_(a°)_, from which a° can be determined by interpolation. The results were shown in Table 4. The last column in this table illustrates the standard deviation σ_Λ_ which was calculated using Eq. (5):

5 $$ \sigma_{\Lambda } = \frac{{\{ \Sigma \, \left( {\Lambda \, _{{{\text{calculated}}}} \, {-} \, \Lambda \, _{{{\text{observed}}}} } \right)^{{2}} \}^{{{1}/{2}}} }}{{\left( {{\text{N }}{-}{ 3}} \right)^{{{1}/{2}}} }}, $$

where N is the number of experimental points.

It can be seen from Table 3 and Fig. 4 that Λo values increases for Sodium *N*,*N*-Dimethyldithiocarbamate from DMF at different temperatures. The values of ao (solvation) also increases in case of using DMF at different temperatures. This means that the ionic equivalent conductance becomes the main factor controlling the extent of ion-pairing.

**Table 3:** The characteristic parameters for Sodium *N*,*N*-Dimethyl from Eq. (4) at pressure 1 atm.

Temperature (°C) ± 0.01	Λ_◦_ (ohm^−1^ equiv^−1^ cm^2^)	J	K_A_	a° (Ǻ)	σ_Λ_
25 °C	72.062 ± 0.21085	1329.1	21.599	7.5	0.0218
30 °C	76.071 ± 0.23985	1492.1	39.173	8.0	0.0169
35 °C	79.854 ± 0.13199	1675.2	57.286	8.5	0.0175
40 °C	84.250 ± 0.25584	1887.6	95.455	9.0	0.0228

In the present work for dilute solutions of Sodium *N*,*N*-Dimethyldithiocarbamate measured in DMF at (25, 30, 35 and 40 °C), the trend was that KA increases in the presence of DMF. It can be seen also from Table 4 that Λ_o_, KA and ao increases, for Sodium *N*,*N*-Dimethyldithiocarbamate in DMF with increasing the temperatures, from 25 to 40 °C, according to ion–dipole interactions between solvents and ions.

**Table 4:** Calculated values of K_2_ and U for Sodium *N*,*N*-Dimethyl dithiocarbamate in Dimethylformamide at 25, 30, 35 and 40°Cand 1 atm.

Temperature (°C) ± 0.01	K_A_	K_1_	K_2_	U
25 °C	21.599	8.140	1.653	0.9758
30 °C	39.173	8.799	3.452	1.4934
35 °C	57.286	9.598	4.969	1.7865
40 °C	95.455	10.493	8.097	2.2079

The trend of K_A_ in the present work was explained in the light of the U term as represented in Eq. (6) ^11^:

6 $$ \ln K_{A} = \ln \left( {\frac{{4\pi Na^{o3} }}{3000}} \right) + \left( {\frac{{e^{2} }}{{a^{o} DkT}}} \right) + U, $$

where

7 $$ U = \left( {\frac{\Delta S}{K}} \right) - \left( {\frac{{E_{s} }}{kT}} \right). $$

(ΔS/k) is the Entropy Boltzmann constant ratio which illustrates the probability of the orientation of the solvent molecules around the free ions and (Es/kT) is an energy relationship which includes the energy of the solvent molecules with respect to the free ions (i.e. ion–dipole interaction) and ion-pairs.

**Figure 5:**
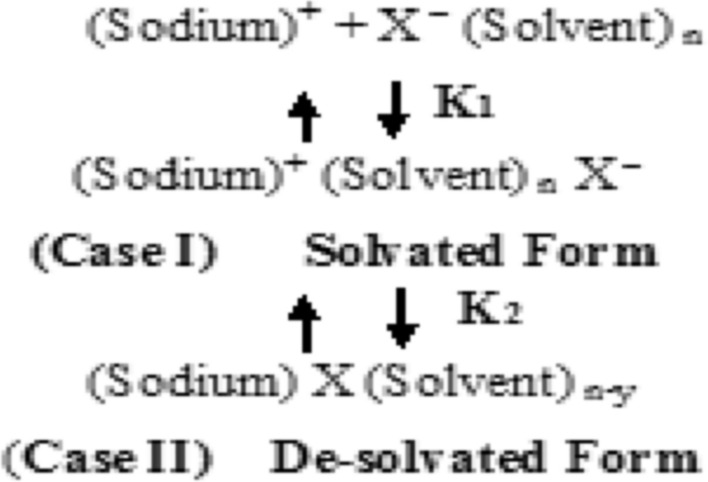
The multiple steps of association^14^.

It can be seen from Table 4 that U term increases with increasing the temperatures for Sodium *N*,*N*-Dimethyldithiocarbamate in DMF from 25 to 40 °C, i.e. the entropy is more predominant than the term of ion–dipole. Finally, solvent separated ion-pair model has been applied^12^. In this model a multiple step association is suggested, i.e. solvent separated and contact ion-pair can be illustrated in the following Fig. 5:

**Table 5:** Calculations of the radii of the ions for Sodium *N*,*N*-Dimethyl dithiocarbamate in Dimethylformamide at 25, 30, 35 and 40 °C and 1 atm (note: (1) ohm^−1^ equiv^−1^ cm^2^ (2) ohm^−1^ equiv^−1^ cm^2^ p).

Temperature (°C) ± 0.01	(1)_**◦**_ **Λ**	λ_◦_^−^ η_◦_ (2)	λ_◦_^−^ (1)	λ_◦_^+^ (1)	R^+^ (A°)	R^−^ (A°)	R^+^ + R^−^ (A°)	a° (A°)
25 °C	72.06	0.332	41.53	30.53	3.355	2.466	5.821	7.5
30 °C	76.07	0.333	44.21	31.86	3.413	2.46	5.873	8
35 °C	79.85	0.335	47.36	32.49	3.567	2.447	6.014	8.5
40 °C	84.25	0.342	51.13	33.12	3.704	2.399	6.103	9

Where** y** is the number of escaping solvent molecules from solvation Thus, the association constant **K**_A_ can be given by the expression:

8 $$ K_{A} = K\sum {\frac{{C_{ion - pairs} }}{{C^{ + }_{sodium} \times C_{{X^{ - }_{{\left( {solvent} \right)}} {}_{n}}} }}} = K_{1} \left( {1 + K_{2} } \right), $$

where K_A_ is obtained from the conductance measurements and since

9 $$ K_{1} = \ln \left( {\frac{{4\pi Na^{o3} }}{3000}} \right), $$

10 $$ K_{2} = \left( {\frac{{e^{2} }}{{a^{o} DkT}}} \right). $$

Then K_2_ can be calculated from Eq. (10). The results compiled in Table 4 indicated that in case of Sodium *N*,*N*-Dimethyl-dithiocarbamate in DMF at different temperatures, K_1_ increases with increasing the temperatures, i.e. ion-pair preferred the solvated form (case I) than the de-solvated form (case II).

##### Radii of ions

The electrostatic radius (R^+^ + R^−^) was given by Stokes' equation:

11 $$ R^{ \pm } = \left( {\frac{{0.{8194 } \times { 1}0^{{ - {8}}} }}{{\lambda_{\rm O}^{ \pm } \eta_{\rm O} }}} \right), $$

where η_o_ is the viscosity of pure solvent and λ_o_^−^ is obtained from the intercept of the straight line, resulting from the plots of Walden product Λ_o_ η_o_ versus the reciprocal of the molecular weight as previously discussed^13^, while λ_o_^+^ for sodium was represented by the value of Λ_o_ of the Sodium *N*,*N*-Dimethyl dithiocarbamate salt.

From the data in Table 5, it could be seen that the values of a° were greater than electrostatic radii (R^+^ + R^−^) obtained from Stokes equation in case of DMF at different temperatures from 25 to 40 °C. This was due to the solvation of ions.

#### Thermodynamic studies of Sodium *N*,*N*-Dimethyldithiocarbamate in DMF from conductance measurements

Thermodynamic parameters (∆H°, ∆G°, ∆S°) and activation energy (∆E_s_) were calculated to explain the limiting equivalent conductance (Λ_0_) and ion association constant (K_A_) of Sodium *N*,*N*-Dimethyl dithiocarbamate in DMF at different temperatures using conductance measurements. Three parameters were evaluated by using Fuoss-Onsager equation. The solvent parameters of DMF at different temperatures (25, 30, 35 and 40 °C) were given in Table 1.

It is evident from Table 6 that, the values of Λ_0_ increase regularly with increasing the temperature for Sodium *N*,*N*-Dimethyl dithiocarbamate salt, indicating less solvation or higher ions mobility in the solvent system studied. This is due to the increase in the results of thermal energy in greater bond breaking and the variation in rotational, vibrational and translational energy of the molecules, which leads to higher frequency and higher ions mobility. In addition, it is clear that the association constant (KA) values increase with increasing the temperature, due to the decrease in dielectric constant of the medium^14^.

**Table 6:** Thermodynamic parameters of Sodium *N*,*N*-Dimethyldithiocarbamate in Dimethylformamide at 25, 30, 35 and 40 °C and 1 atm.

T (K) ± 0.01	K_A_	ΔE_s_^o^ (kJ mol^−1^)	ΔH^o^ (kJ mol^−1^)	ΔG^o^ (kJ mol^−1^)	ΔS^o^ (J mol^−1^ K^−1^)
298	21.59	8.024	75.08	− 7.61	277.50
303	39.17			− 9.24	278.29
308	57.29			− 10.37	277.43
313	95.46			− 11.87	277.79

Since the measurements of the conductance of an ion depend on its mobility, it is reasonable to treat the data of the conductance which similar to the one that employed for the processes taking place with change of temperature (9), i.e.

12 $$ \Lambda_{0} = Ae^{ - \Delta Es/RT} \,{\text{Or}}\,\log \Lambda_{0} = \log A - (\Delta Es/2.303RT), $$

where the symbol A is the frequency factor, R is the constant of the ideal gas and ∆E_S_ is the activation energy of Arrhenius of transport processes.

**Figure 6:**
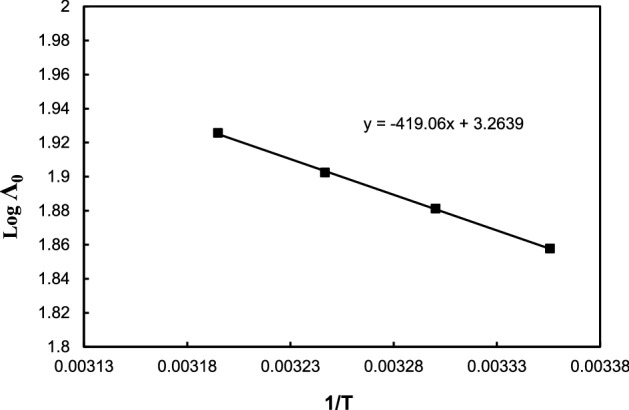
The variation of log Λ_0_ versus 1/T for Sodium *N*,*N*-Dimethyl dithiocarbamate in Dimethylformamide at different temperatures**.**

**Figure 7:**
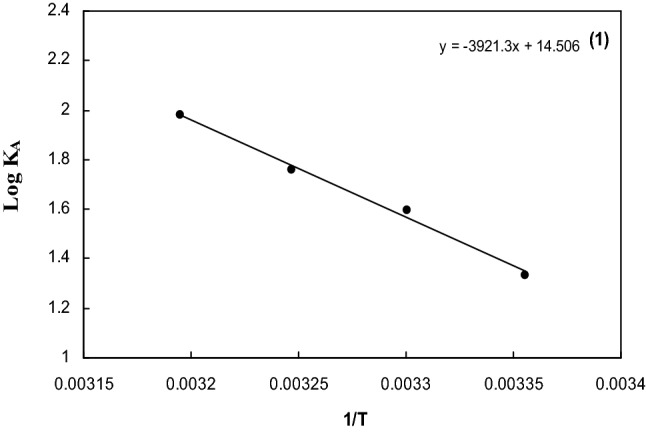
The variation of log K_A_ versus 1/T for Sodium *N*,*N*-Dimethyldithiocarbamate in Dimethylformamide at different temperatures.

The values of ∆E_S_ computed from the slope (− ∆E_S_/2.303R) of the plot of log Λ_0_ versus 1/T and the values recorded in Table 6 and Fig. 5. From the Table 6, the activation energy (∆E_s_) is positive for Sodium *N*,*N*-Dimethyl dithiocarbamate in DMF systems studied. The values increase in case of DMF which indicates the higher mobility of the ions in the solution and hence higher Λ_0_ values. The free energy change ∆G° for the association process is calculated from Eq. (13) ^15^

13 $$ \Delta G^{ \circ } = - RT\ln K_{A} , $$

it is evident from Table 6 and Figs. 6 and 7 that, the free energy change (∆G^o^) values are negative for Sodium *N*,*N*-Dimethyl dithiocarbamate in DMF was studied and this values agree with the values obtained from S.Pura^16^.

This means that the association process is favored over the dissociation process in DMF system. The values of (∆G^o^) become more negative with increasing the temperature. The increase in (∆G°) values for Sodium *N*,*N*-Dimethyl dithiocarbamate salts favors the transfer of the released solvent molecules into bulk solvent and leads to a larger (∆G°) values. The strengthening to the interionic association at higher temperature is largely caused by a decrease in the permittivity of the solvent^17^.

The enthalpy change which is the (heat of association) (∆H°) obtained from the slope of the plot of log K_A_ versus 1/T as shown in Fig. 7. The values of (∆H°) were calculated, where the slope equals (−∆H°/2.303R). The values of (∆H°) are positive which agree with Dash et.al.^14^.

The positive values of (∆H°) for Sodium *N*,*N*-Dimethyl dithiocarbamate salt shows that the association processes are endothermic in nature. Positive values and high values of (∆H°) attributed to the interaction between ions^17^.

The entropy change (∆S°) was calculated, from Gibbs–Helmholtz Eq. (14):

14 $$ \Delta G^{ \circ } = \Delta H^{ \circ } - T\Delta S^{ \circ } . $$

The values of (∆S°) are positive which agree with Dash et al.^13^ the positive values of (∆S°) for Sodium *N*,*N*-Dimethyl dithiocarbamate salt indicate the randomness of ions in all solvent systems studied. As presented in Table 6, values of (∆S°) were positive because of the decrease in the solvation of ion-pair compared to that of the free ion which agree with Bag et al.^18^. This may be attributed to increase in the degree of freedom upon association, mainly due to the release of solvent molecules this values agree with the values obtained^19–22,25–33^.

The main factors, which govern the standard entropy of ion association of electrolytes, are.

I. The ions size and shape.
II. Charge density on ions.
III. The solvent molecules electrostriction around the ions and
IV. The solvent penetration of the molecules inside the space of ions^23,24^.

## Conclusions

The following conclusions arise from the work described here in:

1. The activation energy (∆Es) is positive for Sodium *N*,*N*-Dimethyl dithiocarbamate in DMF systems and is high due to the higher mobility of the ions in the solution and hence higher Λ_0_ values
2. The free energy change (∆G°) values is negative and this means that the association process is favored over the dissociation process in DMF system.
3. The values of (∆G°) are more negative with increasing the temperature.
4. The enthalpy change (heat of association) (∆H°) is positive and this means that the association processes are endothermic in nature and attributed to the interaction between ions.
5. The positive values of (∆S°) indicates the randomness of ions in all solvent systems studied due to the decrease of degree of solvation of ion-pair compared to that of the free ion. This may be attributed to the increasing in degree of freedom upon association.

## Data Availability

The datasets used and analyzed during the current study are available from the corresponding author on reasonable request.

## List of symbols

K_A_: The association constant
y: The number of escaping solvent molecules from solvation
(ΔS/k): The Entropy Boltzmann constant ratio which illustrates the probability of the orientation of the solvent molecules around the free ions
(E_s_/kT): An energy relationship
r: The resistance of solution
C: Concentration (equiv. L^−^^1^)
Λ: Equivalent conductance (ohm^−^^1^ equiv^−^^1^ cm^2^)
Specific conductance of solution (ohm^−^^1^ cm^−^^1^)
d: The absolute density (g L^−^^1^)
η: The viscosity
D: The dielectric constant
The specific conductance of the pure solvents at different temperatures
Λ_o_: The equivalent conductance at infinite dilution
η_o_: The viscosity of pure solvent
R^+^ + R^−^: Electrostatic radii
η_o_: The viscosity of pure solvent
A: The frequency factor
R: The ideal gas constant
∆E_S_: Arrhenius activation energy of transport processes
∆G°: The free energy change
∆H°: The enthalpy change (heat of association)
∆S°: The entropy change
