# Author Correction: Electrical conductivity and thermodynamic studies on Sodium Dimethyldithiocarbamate in non aqueous solvents Dimethylformamide (DMF), at different temperatures

**DOI:** 10.1038/s41598-023-28705-x

**Published:** 2023-01-26

**Authors:** W. A. Hammad, N. H. El-Hammamy, M. H. Morshidy, Kholood Alkamis, M. A. Darweesh

**Affiliations:** 1 Faculty of Engineering, Tanta University, Tanta, Egypt; 2 Faculty of Science, Alexandria University, Alexandria, Egypt; 3 Tabuk University, Tabuk, Saudi Arabia

Correction to: *Scientific Reports* 10.1038/s41598-022-18849-7, published online 17 September 2022

The original version of this Article contained an error in the spelling of the author Kholood Alkamis which was incorrectly given as Kh. M. Alkhamis.

The original Article has been corrected.

